# High-resolution RNA allelotyping along the inactive X chromosome: evidence of RNA polymerase III in regulating chromatin configuration

**DOI:** 10.1038/srep45460

**Published:** 2017-04-03

**Authors:** Ru Hong, Bingqing Lin, Xinyi Lu, Lan-Tian Lai, Xin Chen, Amartya Sanyal, Huck-Hui Ng, Kun Zhang, Li-Feng Zhang

**Affiliations:** 1 School of Biological Sciences, Nanyang Technological University, 60 Nanyang Drive 637551, Singapore; 2 Genome Institute of Singapore, 138672, Singapore; 3 Division of Mathematical Sciences, School of Physical and Mathematical Sciences, Nanyang Technological University, 21 Nanyang Link 637371, Singapore; 4 Department of Bioengineering, University of California at San Diego, La Jolla, CA 92093, USA

## Abstract

We carried out padlock capture, a high-resolution RNA allelotyping method, to study X chromosome inactivation (XCI). We examined the gene reactivation pattern along the inactive X (Xi), after *Xist* (X-inactive specific transcript), a prototype long non-coding RNA essential for establishing X chromosome inactivation (XCI) in early embryos, is conditionally deleted from Xi in somatic cells (Xi^∆*Xist*^). We also monitored the behaviors of X-linked non-coding transcripts before and after XCI. In each mutant cell line, gene reactivation occurs to ~6% genes along Xi^∆*Xist*^ in a recognizable pattern. Genes with upstream regions enriched for SINEs are prone to be reactivated. SINE is a class of retrotransposon transcribed by RNA polymerase III (Pol III). Intriguingly, a significant fraction of Pol III transcription from non-coding regions is not subjected to *Xist*-mediated transcriptional silencing. Pol III inhibition affects gene reactivation status along Xi^∆*Xist*^, alters chromatin configuration and interferes with the establishment XCI during *in vitro* differentiation of ES cells. These results suggest that Pol III transcription is involved in chromatin structure re-organization during the onset of XCI and functions as a general mechanism regulating chromatin configuration in mammalian cells.

XCI is an early developmental event for balancing the X-linked gene dosage between males and females^1^. *Xist* is a prototype long non-coding RNA (lncRNA) essential for the establishment of XCI. At the onset of XCI, the X-linked *Xist* gene becomes allele-specifically expressed from one X in each female cell and the RNA transcripts spread along the chromosome territory in *cis* recruiting other silencing factors to condense the chromatin structure to achieve the chromosome-wide gene silencing. The inactive X (Xi) forms a distinctive heterochromatin structure known as the Barr body. The silencing status of Xi is tightly maintained in somatic cells by a poorly understood mechanism^2^. XCI in somatic cells remains largely intact even upon conditional *Xist* deletion from Xi^3^,^4^,^5^. Based on allele-specific RT-PCR results from a small sampling of selected genes, gene reactivation along Xi^∆*Xist*^ was believed to be rare and sporadic^3^. However, it was unclear how many genes are reactivated along Xi^∆*Xist*^ and whether the gene reactivation pattern is truly sporadic. To comprehensively profile the rare gene reactivation along Xi^∆*Xist*^, a high-resolution RNA allelotyping method is preferable to direct RNA-Seq. In direct RNA-seq, the majority of the sequencing power is unavoidably wasted on autosomal genes and sequences without DNA polymorphism for allelotyping.

The vast majority of the mammalian genome does not carry coding sequences for proteins^6^,^7^. These non-coding regions are enriched with repetitive DNA elements and are sometimes referred to as “junk DNA” without known biological functions. Extensive transcriptional activity can be detected along the non-coding DNA regions and a growing list of lncRNAs is being identified^8^. Many lncRNAs are involved in epigenetic regulation. It is interesting to study the behaviors of X-linked non-coding transcripts during XCI. Is there another non-coding RNA with a similar expression profile as *Xist*, which is biallelically expressed at low levels in undifferentiated cells and allele-specifically expressed from Xi in somatic cells ? Are non-coding transcripts subjected to *Xist*-mediated transcriptional silencing ? If so, are they reactivated along Xi^∆*Xist*^ ? Again, a high-resolution RNA allelotyping method is required to answer these questions.

## Results

### Padlock SNP capture

**Figure 1:**
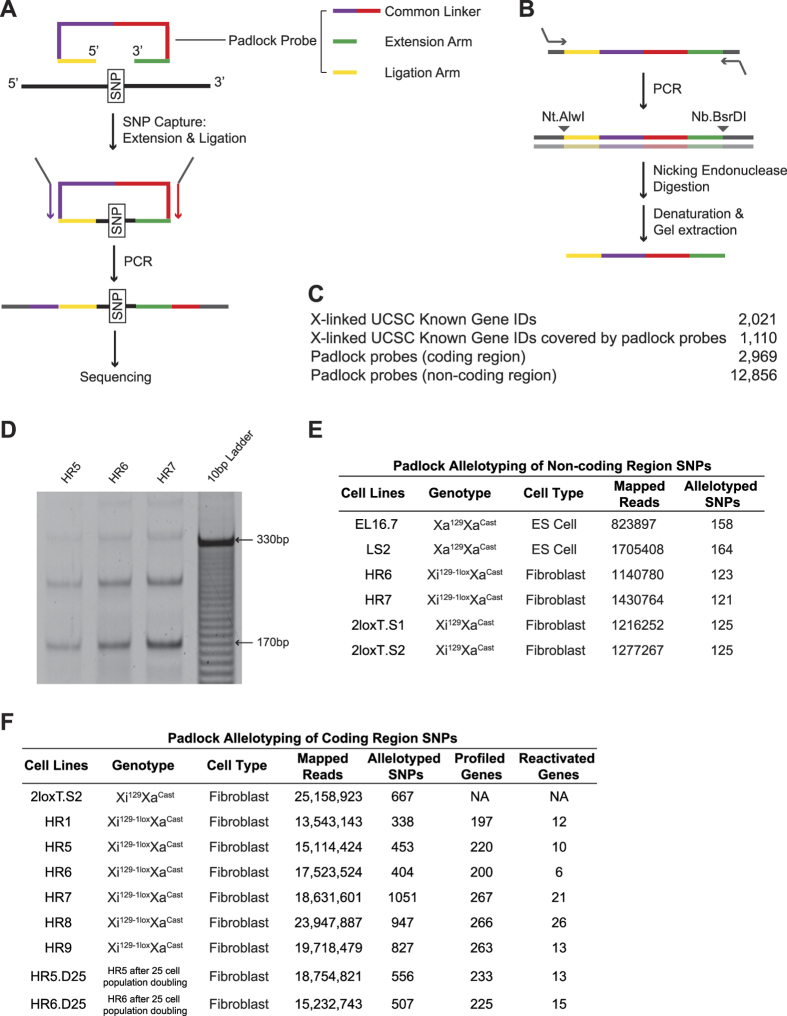
Padlock SNP capture. (**A**) Scheme of SNP capture using padlock probes. (**B**) Diagram of padlock probe library amplification. (**C**) Summary of padlock probe library design. (**D**) PCR amplification of the circular DNA molecules generated in padlock probe SNP capture. The DNA bands showed the expected amplicon sizes. The ~175 bp band was subjected to Solexa sequencing. The ~255 bp, ~345 bp and ~435 bp bands correspond to PCR extension around the circular DNA templates (a unique feature of the padlock capture products) 2, 3 and 4 times. (**E**,**F**) Summary of the Solexa sequencing data.

Padlock SNP capture is a RNA allelotyping method^9^ optimal to study XCI. A library of short DNA oligos, called “padlock probes”, helps to concentrate the sequencing power onto a selected pool of SNPs (Fig. 1A,B and D) to achieve high-throughput and high-resolution RNA allelotyping.

We established two padlock libraries. Each padlock probe targets one SNP. The padlock library for protein-coding genes targets 2,969 SNPs covering ~55% of X-linked genes (Fig. 1C). The padlock library for non-coding regions targets 12,856 SNPs randomly selected from non-coding regions (Fig. 1C).

All female mouse cell lines used in this study carry Xs from two different mouse strains, the 129 strain and the *Mus musculus castaneus* (CAST/Ei) strain (Cast). A female mouse embryonic fibroblast (MEF) cell line (2loxT)^3^ with two loxP sites inserted into the *Xist* gene on the X^129^ allele (X^129-2lox^X^Cast^)^5^ was used to generate the mutant cell lines. Through limiting dilution, six mutant cell lines (HR1, HR5-9) were established, in which X^129^ was non-randomly inactivated and *Xist* was allele-specifically deleted from Xi^129^ (Xi^129-1lox^Xa^Cast^) (Supplementary Figure S1). Furthermore, HR5 and HR6 were subjected to 25 cell population doublings in a long-term cell culture to establish two cell populations (HR5.D25 and HR6.D25). Two control MEF lines (2loxT.S1 and 2loxT.S2) with the genotype Xi^129^Xa^Cast^ were generated from 2loxT cells through limiting dilution. Two female ES cell lines (EL16.7 and LS2) with the genotype Xa^129^Xa^Cast^ were also included to represent the pre-XCI cells.

### Gene reactivation pattern along Xi^∆*Xist*^

**Figure 2:**
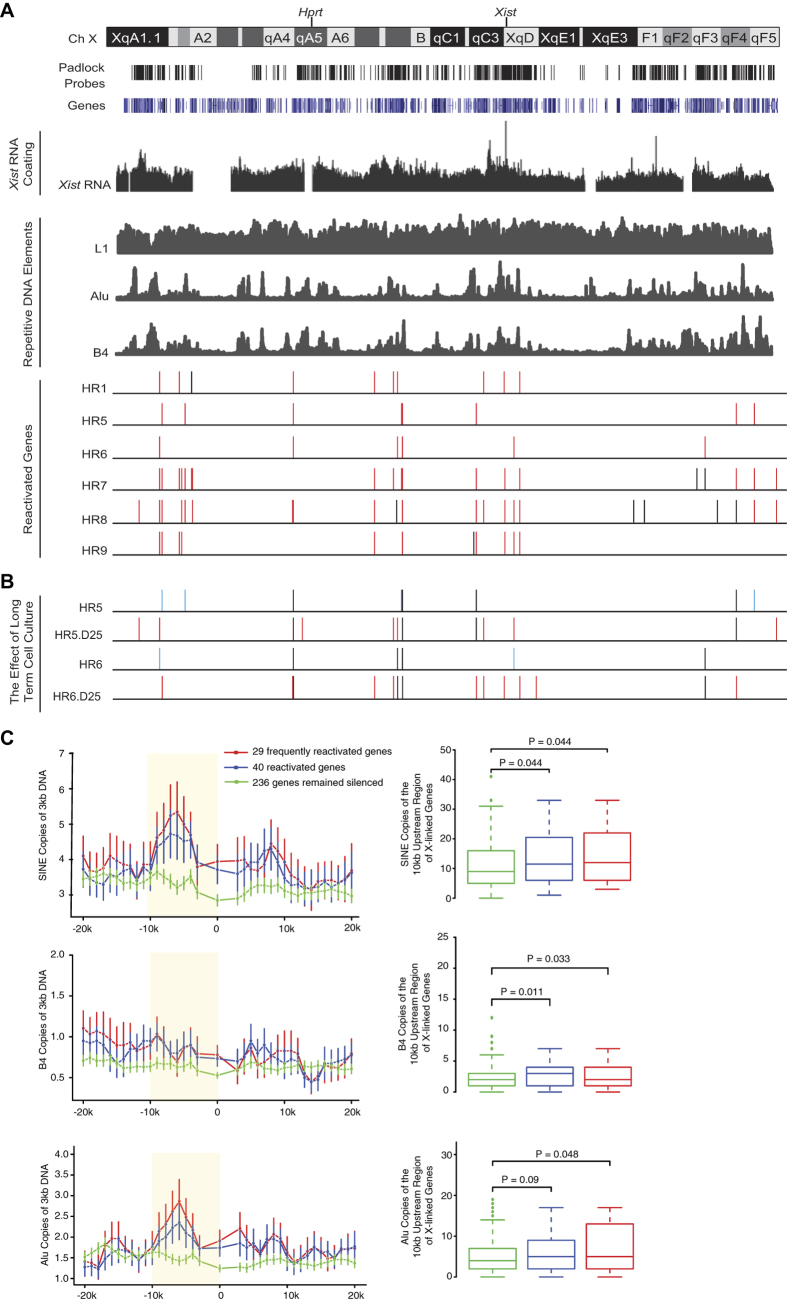
The gene reactivation pattern along the Xi^∆*Xist*^ allele. (**A**) The alignment of genes, padlock probes, reactivated genes and other genetic features along the mouse X chromosome. Each reactivated gene is represented by a bar positioned at the gene’s corresponding location along the mouse X chromosome. Genes reactivated in at least 2 out of the 8 mutant samples are marked in red. The positions of *Xist* and *Hprt* genes are marked. The distribution of three repetitive DNA elements (L1, Alu and B4) along the mouse X chromosome is shown. The *Xist* RNA enrichment along the Xi in mouse lung fibroblast (MLF) was re-plotted from the data generated by previous studies^15^. (**B**) The effect of long-term cell culture on two Xi^129-ΔXist^Xa^Cast^ mutant cell lines. The genes, which were reactivated during the long-term cell culture, are labeled in red. The genes, which were silenced during the long-term cell culture, are labeled in blue. (**C**) Distribution of SINE elements along the X-linked genes. The 20 kb upstream and the 20 kb downstream regions of each X-linked gene were scanned by a sliding window of 3 kb with a step size of 1 kb. The average copy number of a repetitive element within a 3 kb intronic region of a gene is used to represent the density of the repetitive element within the gene body. Data are shown as mean ± S.E.M. Box plots show the of copy number of a repetitive element within the 10 kb DNA region upstream of X-linked genes.

To study the gene reactivation along Xi^∆*Xist*^, we carried out high-resolution RNA allelotyping on 9 RNA samples (Fig. 1F). 13–25 million mapped sequencing reads were obtained from each sample. 11–35% of the targeted SNPs and 18–25% of the X-linked genes were allelotyped in each sample (Fig. 1F). The results confirmed that low-level gene reactivation occurred along Xi^∆*Xist*^ (Figs 1F and 2A). On average, ~6% of the X-linked genes were reactivated in each mutant cell line. To identify the reactivated genes, we calculate the gene’s allelotype as “Reads^129/^Reads^Cast^”. For a reactivated gene, its allelotype in the mutant cells should be at least 3 folds of its allelotype in the control. Moreover, a reactivated gene should be biallelically expressed in the mutant cells (0.01 < allelotype < 100). Among the 276 X-linked genes allelotyped, 40 genes (~14%) were reactivated in at least one mutant. 29 genes, which were reactivated in 2 or more mutants, were named as “frequently reactivated genes” (Fig. 2A and Supplementary Table S1). 8 constitutive escapees^10^ and 3 facultative escapees^11^ of XCI also showed significantly increased expression along Xi^∆*Xist*^ and therefore were included as the reactivated genes (Supplementary Table S1).

The repetitive DNA element, LINE-1 (L1), has been speculated as the “way station” DNA element facilitating the *Xist* RNA to spread along its host chromosome^12^. To study the correlation between the gene reactivation pattern and the distribution of repetitive DNA elements, we calculated the Spearman correlation coefficient between the distribution of the reactivated genes and 40 different families of repetitive DNA elements (Supplementary Table S2). The gene reactivation pattern is positively correlated with SINE element distribution, for example Alu (Spearman’s correlation = 0.62) and B4 (Spearman’s correlation = 0.61). In the mouse, the class of SINE retrotransposon contains four families, Alu, B2, ID and B4^13^. In addition to SINEs, the gene reactivation pattern is also positively correlated with gene density and *Xist* RNA coating^14^,^15^ (Spearman’s correlation = 0.54). The distribution of L1 element is not correlated with the gene reactivation pattern (Spearman’s correlation = −0.14).

It is possible that the positive correlation between gene reactivation and SINE density is a mere consequence of the high gene density in gene-rich islands. Gene reactivation is more likely to occur along gene-rich regions, and these regions are known to be enriched for *Xist* RNA coating^14^,^15^ and SINEs^7^. However, arguing against this possibility, examination on SINE distribution along individual genes showed that SINEs are significantly enriched along the upstream regions of the reactivated genes compared to the genes remained silenced (Fig. 2C, the yellow-highlighted region and Supplementary Figure S2). Analyzing the list of reactivated genes, which excludes the constitutive escapees and the genes biallelically expressed in the control cell line, yielded similar results (Supplementary Figure S3). In summary, the gene reactivation along Xi^∆*Xist*^ is rare but not sporadic. Curiously, genes with SINE enrichment along their upstream regions are prone to be reactivated.

Long-term cell culture did not enlarge the epigenetic lesion along Xi^∆*Xist*^. The gene reactivation level remained low in HR5.D25 and HR6.D25 (Figs 1F and 2B). The long-term cell culture could either reactivate a silenced gene or silence a reactivated gene (Fig. 2B), suggesting that the epigenetic status of Xi^∆*Xist*^ is not duplicated with 100% fidelity during each cell cycle. However, we could not rule out the possibility that some of the gene reactivation events were functional such that cells carrying these reactivation events were gradually eliminated during the long-term cell culture, although no obvious cell death was observed in our experiments.

### A significant fraction of non-coding transcription is not subjected to *Xist*-mediated silencing

**Figure 3:**
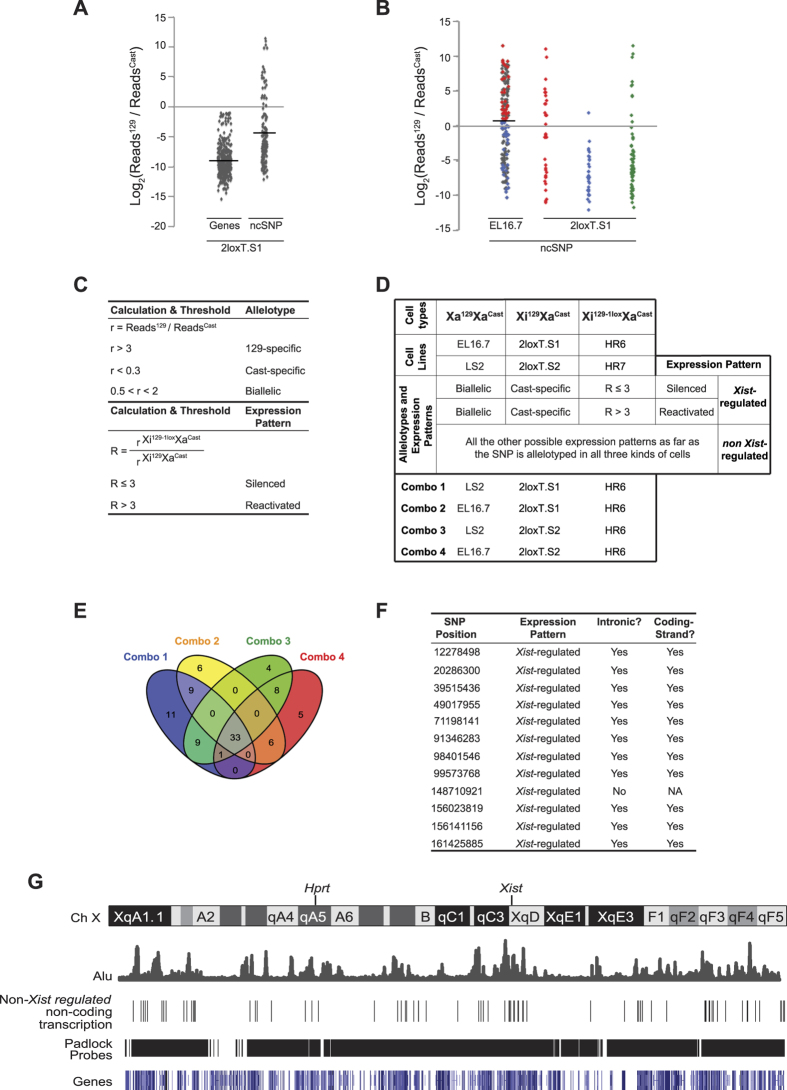
Non-coding transcription along the Xi is largely independent from *Xist*-mediated gene silencing. (**A**) The allelotype of X-linked ncSNPs and X-linked genes in 2loxT.S1 cells. (**B**) The allelotype of X-linked ncSNPs in EL16.7 and 2loxT.S1 cells. The SNPs only allelotyped in one cell type are labeled in gray (EL16.7) and green (2loxT.S1). The SNPs allelotyped in both cell types are labeled in red or blue. The red dots represent the SNPs with an above-average allelotype score in EL16.7. The blue dots represent the SNPs with a below-average allelotype score in EL16.7. The data from 2loxT.S1 cells is grouped into three columns for visualizing the allelotype changes of the ncSNPs before and after XCI. (**C**) Calculations and thresholds used in analyzing the data of padlock capture of X-linked non-coding transcriptions. (**D**) The definitions of “expression pattern”, which are used to describe the allele-specific expression profile of an X-linked non-coding SNP. Cell lines of three different genotypes were used in the study. Two cell lines were selected for each genotype. The data from different cell lines were combined in four different ways to test the consistency of the experiments. (**E**) A Venn diagram showing the non-coding SNPs with “non-*Xist* regulated” expression pattern from the data analysis of four different combinations of data sets. (**F**) Non-coding SNPs with “*Xist*-regulated” expression pattern from the data analysis of four different combinations of data sets. (**G**) The distributions of genes, Alu elements, padlock probes targeting non-coding regions and the “non-*Xist* regulated” non-coding transcription activities detected along the mouse X chromosome.

To study the behaviors of non-coding transcripts during XCI, three cell types were included (Xa^129^Xa^Cast^, Xi^129^Xa^Cast^ and Xi^129-1lox^Xa^Cast^) (Figs 1E and 3). Two different cell lines were selected for each cell type (Fig. 3D). The padlock library targets 12,856 SNPs randomly selected from intronic and intergenic regions (Fig. 1C). Since a comprehensive non-coding transcription profile is not available, one padlock probe targeting a non-coding SNP along the sense strand of X chromosome and one padlock probe targeting the antisense strand were randomly selected from every 5 kb region. To avoid misleading results generated by highly repetitive DNA sequences, only the sequencing reads with single best alignment position along the mouse genome were considered in data analysis. On average, ~130 non-coding SNPs were allelotyped in each cell line and each SNP was allelotyped by ~9,000 reads (Fig. 1E).

Strikingly, completely different from X-linked genes, which are exclusively expressed from X^cast^ in the control fibroblasts (Xi^129^Xa^Cast^), the allelotype of non-coding transcription showed a high degree of allele bias against either X^cast^ or X^129^ (Fig. 3A). Comparing the allelotype of ncSNPs in one female ES cell line (EL16.7) and one control fibroblast cell line (2loxT.S1) showed that a significant fraction of non-coding transcription is not subjected to *Xist*-mediated transcriptional silencing (Fig. 3B). The transcription activities across many ncSNPs were resistant to *Xist*-mediated transcriptional silencing because the transcription along X^129^ was not silenced by *Xist*. Meanwhile, the transcription activities across many other ncSNPs were un-related to *Xist*-mediated transcriptional silencing. For example, the transcription along X^129^ was silenced before and after XCI.

To further analyze the data, we define two terms, “allelotype” and “expression pattern”. A SNP’s allele-specific expression status (“Reads^129/^Reads^Cast^”) is the SNP’s allelotype, which includes “129-specific”, “cast-specific” and “biallelic” (Fig. 3C). Moreover, a SNP’s allelotype could also be “undetermined” or “undetected” depending on the thresholds used in data analysis (Fig. 3C). If a SNP is allelotyped in all three cell types (Xa^129^Xa^Cast^, Xi^129^Xa^Cast^ and Xi^129-1lox^Xa^Cast^), its “expression pattern” is then determined (Fig. 3D). If a transcription activity is subjected to *Xist*-mediated gene silencing (“*Xist*-regulated”), the SNP’s allelotype should be biallelic in Xa^129^Xa^Cast^ cells and cast-specific in Xi^129^Xa^Cast^ cells (Fig. 3D). All other expression patterns are considered as “non *Xist*-regulated”, which means the transcription activity is not subjected to *Xist*-mediated gene silencing. If a transcription activity is “*Xist*-regulated”, its expression pattern can be further determined as “silenced” (remaining silenced in Xi^129-1lox^Xa^Cast^ cells) or “reactivated” (reactivated in Xi^129-1lox^Xa^Cast^ cells) (Fig. 3D). In our study, we did not detect any non-coding transcription with the expression pattern similar to *Xist*, which is biallelic in Xa^129^Xa^Cast^ cells and 129-specific in Xi^129^Xa^Cast^ cells.

Since two different cell lines were included to represent each cell type, we analyzed the data in four different combinations (“Combo”) of cell lines (Fig. 3D). Most of the SNPs showed “non *Xist*-regulated” expression pattern. The identified “non *Xist*-regulated” SNPs are highly consistent among the four combinations of data analysis (Fig. 3E). Only 12 SNPs were *Xist*-regulated (Fig. 3F). Interestingly, all except one of the 12 SNPs are located within introns and along the coding strands of X-link genes. Therefore, the “*Xist*-regulated” expression pattern detected from these 11 SNPs may reflect the expression pattern of unspliced nascent transcripts of X-linked genes. This result further confirmed the sensitivity and consistency of the experiments. To avoid subjectivity of threshold selection involved in the data analysis, the data were re-analyzed using a different set of thresholds (Supplementary Figure S4). The two rounds of data analysis results are highly consistent. In conclusion, transcription along the non-coding regions of the X chromosome shows a high degree of allelic bias. A significant fraction of the non-coding transcription is not subjected to *Xist*-mediated transcriptional silencing.

### Role of Pol III transcription in regulating chromatin configuration

**Figure 4:**
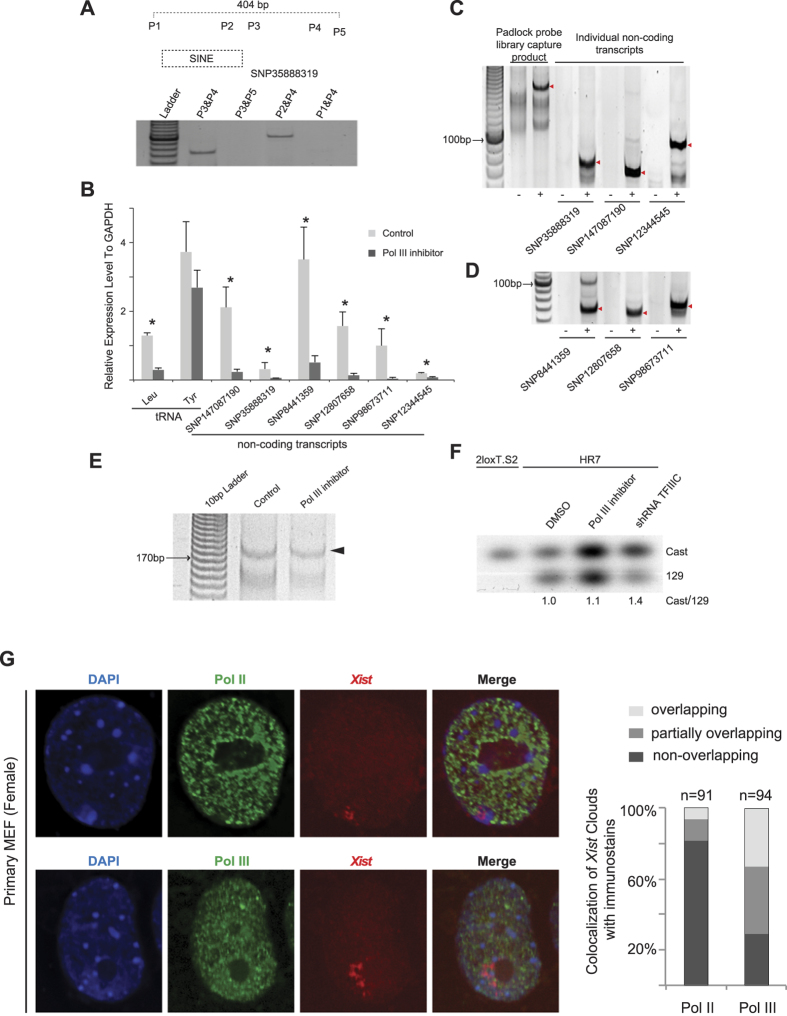
Pol III transcription from non-coding regions is involved in regulating the chromatin architecture. (**A**) RT-PCR to check the range of the non-coding transcription across SNP35888319 in 2loxT.S2 cells. (**B**) Quantitative RT-PCR to check the expression level of the non-coding transcription before and after 2loxT.S2 cells were treated with a Pol III inhibitor. Data are shown as mean ± S.E.M of biological triplicate. The statistical analysis used is the Student’s *t*-test. One asterisk indicates *P*-values smaller than 0.05. tRNA precursors (Pol III genes) serve as positive controls. (**C**,**D**) Reverse transcription followed by padlock capture or PCR to detect the non-coding RNA transcripts and the corresponding -RT controls. The red arrowheads indicate the PCR fragments with the expected product size in each reaction. (**E**) Padlock capture of the X-linked non-coding transcripts from 2loxT.S2 cells treated with the Pol III inhibitor. The padlock capture was carried out using the padlock probe library. The arrowhead indicates the padlock capture products with the expected size of ~175 bp. The amount of cDNA used in padlock capture was normalized according to a separate quantitative RT-PCR reaction on *β-actin*. (**F**) Allele-specific RT-PCR followed by southern blotting on *Med14* (uc009sqz.1), a reactivated gene along the Xi^129–ΔX*ist*^ allele in HR7 cells. (**G**) Immuno-RNA FISH to detect RNA polymerases and the *Xist* RNA. Immunofluorescence was performed using mouse monoclonal antibodies against RNA polymerase II (Pol II) and RNA polymerase III (Pol III) with a secondary antibody conjugated with FITC (green). Immunostaining was followed by RNA FISH with Cy3-labeled probe (red) to detect the *Xist* RNA. Images shown are deconvolved images from a single z-section. DNA was counterstained with DAPI (blue).

In total, 92 SNPs showed “non *Xist*-regulated” expression pattern in the four combinations of data sets (Fig. 3E). Among them, 7 SNPs were detected as “*Xist*-regulated” in one data set and as “non *Xist*-regulated” in others. 85 SNPs were detected only as “non *Xist*-regulated” (Fig. 3E and Supplementary Table S3). The distribution of these 85 SNPs is again positively correlated with SINE distribution (Fig. 3G) (Spearman’s correlation = 0.72). SINEs are retrotransposons transcribed by Pol III^7^. We tested whether some of the detected non-coding transcriptions are directly from SINEs. By reverse transcription, we narrowed down the transcription range across SNP35888319 to a ~400 bp region, a length consistent with Pol III transcription (Fig. 4A). The region contains a putative SINE element (Fig. 4A). The transcription was inhibited by a Pol III specific inhibitor (ML-60218)^16^ (Fig. 4B). These results suggest that the non-coding transcript detected across SNP35888319 is a transcript of SINE. In addition to SNP35888319, the transcription across five other non-coding SNPs was also validated and found to be repressed by the Pol III inhibitor (Fig. 4B). The expression levels of these non-coding RNAs were similar to the tRNA precursors (Fig. 4B). –RT controls showed that the detected RT-PCR products were not due to DNA contamination (Fig. 4C and D). Two Pol II genes (*Gapdh and β-actin*) were used as controls and confirmed that the inhibitor specifically inhibits Pol III genes (Supplementary Figure S5). Furthermore, the total amount of the non-coding transcripts captured by the padlock probe library was reduced by Pol III inhibition (Fig. 4E). These results confirm that a significant fraction of the non-coding transcription detected by the padlock probes is driven by Pol III. Consistent with the RT-PCR result, immuno-RNA FISH showed that, in contrast to Pol II which is largely excluded from the chromosome territories covered by *Xist* clouds, ~71% of *Xist* clouds were overlapping or partially overlapping with Pol III immunostain (Fig. 4G), suggesting on-going Pol III transcription along the Xi.

Since X-linked genes with SINE enrichment along its upstream region are prone to be reactivated along Xi^∆*Xist*^, we carried out experiments to study whether Pol III inhibition affects the gene reactivation status along Xi^∆*Xist*^. The transcription across the non-coding SNP (12344545), which was repressed by the Pol III inhibitor (Fig. 4B), is located upstream of a gene *Med14* (uc009sqz.1), which was reactivated in HR7 cells (Fig. 4F). We tested whether Pol III inhibition could re-silence the reactivated gene. As shown in Fig. 4F, the reactivated transcription along the 129 allele was slightly repressed when the cells were treated by the Pol III inhibitor. The repressive effect is more obvious in the cells transfected with shRNA against TFIIIC (transcription factor for polymerase III C).

**Figure 5:**
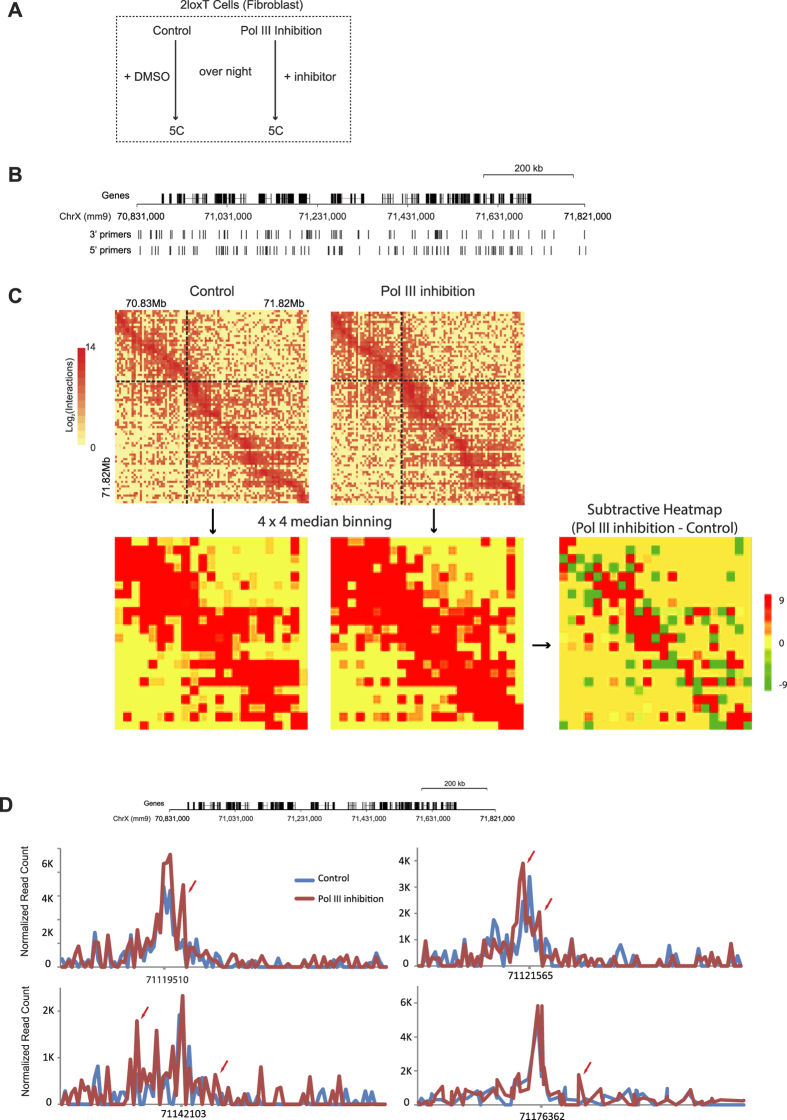
Pol III inhibition causes chromatin conformation change. (**A**) The experimental design. (**B**) The ~1 mb region along the mouse X chromosome selected for 5 C analysis. (**C**) Heatmaps showing all the chromatin interactions detected in each sample. The two dashed lines drawn in the control sample heatmap mark the boundaries of the two topologically associating domains (TAD) detected in the control sample. To show the difference of chromatin interaction between the two samples, a subtractive heatmap was generated by subtracting the interaction count of the control sample from the interaction count of the Pol III inhibition sample. (**D**) Chromatin interaction profiles of four selected anchor points.

Based on these results, we speculated that Pol III transcription along non-coding regions might be involved in regulating chromatin configuration. We carried out 5 C (Carbon-Copy Chromosome Conformation Capture) (Fig. 5A) on a ~1 mb region (Fig. 5B) selected from the X chromosome around the *Mecp2* gene to test whether Pol III inhibition causes chromatin structural change. Pol III inhibition caused detectable change in chromatin configuration (Fig. 5C and D). Two topologically associating domains can be recognized in the heatmap of the control sample (Fig. 5C). Pol III inhibition generated more chromatin interactions especially around the boundaries of the two topologically associating domains. These results show that Pol III inhibition affects chromatin configuration.

### Pol III inhibition impairs XCI *in vitro*

**Figure 6:**
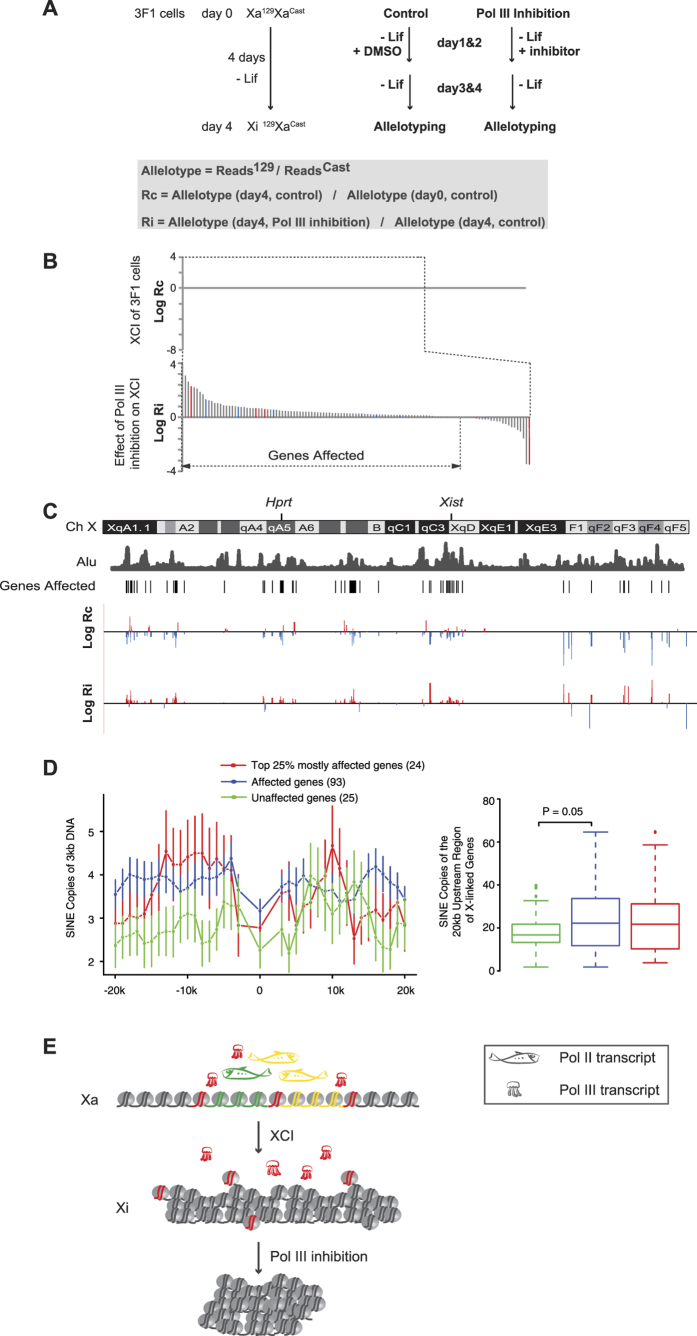
Pol III inhibition impairs XCI *in vitro*. (**A**) The experimental design. (**B**) The effect of Pol III inhibition on the XCI of 3F1 cells *in vitro*. X-linked genes were sorted according to their corresponding values on the y-axis. The constitutive escapees^10^ of XCI are highlighted in red. The facultative escapees^11^ of XCI are highlighted in blue. Note: Genes are sorted along the x-axis according to Rc or Ri values. Readers are referred to panel C for the corresponding values of Rc and Ri of each gene. (**C**) The distribution pattern of the X-linked genes affected by Pol III inhibition. The Log Rc and Log Ri values of the X-linked genes are plotted along the X chromosome at the corresponding position of each gene. (**D**) Distribution of SINE elements along the X-linked genes. The 20 kb upstream and the 20 kb downstream regions of each X-linked gene were scanned by a sliding window of 3 kb with a step size of 1 kb. The average copy number of a repetitive element within a 3 kb intronic region of a gene is used to represent the density of the repetitive element within the gene body. Data are shown as mean ± S.E.M. Box plots show the of copy number of SINE elements within 20 kb upstream of X-linked genes. (**E**) A model of Pol III transcription from noncoding regions regulating the chromatin configuration.

Global chromatin structure reorganization is involved in establishing XCI. To study the role of Pol III transcription in XCI, we allelotyped X-linked gene expression using padlock capture in 3F1 cells. 3F1 is a mutant female ES cell line (Xa^129^Xa^Cast^)^17^, in which XCI is carried out in a non-random manner due to the genetically disrupted “choice” step during XCI. The “preemptive choice” mutant phenotype of 3F1 cells not only causes non-random inactivation of the X^129^ allele (Fig. 6A), which enables us to use RNA allelotyping to monitor XCI, but also expedites the XCI process *in vitro*, which helps to prevent prolonged Pol III inhibition in the experiment. We carried out *in vitro* differentiation of 3F1 cells for 4 days. The cells were treated with the Pol III inhibitor during the first two days of *in vitro* differentiation (Fig. 6A). *Xist* RNA FISH shows that Pol III inhibition did not affect the up-regulation of *Xist* expression in differentiating ES cells (Supplementary Figure S6). From the control sample, 169 genes were allelotyped and XCI was detected on 118 genes (70%) (Fig. 6B). Among the 118 genes, the XCI of 93 genes (79%) (Supplementary Table S4) were delayed or impaired by Pol III inhibition (Fig. 6B and C). The distribution of the 93 genes affected by Pol III inhibition is positively correlated with SINEs (Fig. 6C) (Spearman’s correlation = 0.72). SINE elements are clearly enriched along the upstream regions of the 93 affected genes comparing to the 25 unaffected genes (Fig. 6D). Taken together, these results show that Pol III inhibition delays the establishment of XCI during *in vitro* differentiation of ES cells and genes with SINE elements enriched along their upstream regions are more sensitive to Pol III inhibition. These results argue against the possibility that the impaired XCI was caused by a general effect of Pol III inhibition on cell proliferation and suggest a functional role of Pol III transcription in regulating chromatin configuration.

## Discussion

Our study confirms that XCI is largely maintained along Xi^∆*Xist*^ in somatic cells. Gene reactivation occurs rarely along Xi^∆*Xist*^. The mechanism of XCI maintenance awaits elucidation. Among the reactivated genes identified in this study, no genes were reactivated in all the 8 mutant samples. This result suggests that the chromatin configuration of Xi^∆*Xist*^ is misregulated with a certain degree of randomness. Meanwhile, the functional effect of misregulated gene expression along Xi^∆*Xist*^ on cell survival should not be ruled out. Our study also provides new insights on escapees of XCI. Although the transcription of an escapee is not completely silenced along Xi, the transcription level is often repressed by *Xist*-mediated gene silencing (Fig. 6B) and often de-repressed along Xi^∆*Xist*^ (Supplementary Table S1).

Although gene reactivation occurs rarely along Xi^∆*Xist*^, the gene reactivation pattern is not sporadic. Genes with SINE enrichment along its upstream region are prone to be reactivated. The list of Pol III transcribed genes in mammalian genomes is long and growing^18^. All Pol III transcripts are short RNAs, for example tRNAs and SINEs. It is reasonable to assume that some of the Pol III transcriptions are immune to *Xist*-mediated transcriptional silencing, because they are transcribed from individual nucleosomes or short DNA regions, which are located at special positions “impervious” to or independent of the influence of the neighboring higher order chromatin structure (Fig. 6E). *Xist*-mediated transcriptional silencing depends on a compact higher order chromatin structure. Therefore, a fraction of Pol III transcription along non-coding regions is not subjected to *Xist*-mediated silencing (Fig. 6E). Furthermore, our study suggests that Pol III transcription is involved in a general mechanism of regulating local chromatin configuration. Upon Pol III inhibition, local chromatin configuration may become more compact, more rigid and less cooperative in a global chromatin structure reorganization process, such as XCI. On the other hand, pol III transcription along non-coding regions may cause the local chromatin configuration “impervious” to the influence of the neighboring higher order chromatin structure. In consequence, pol III transcription along non-coding regions may either function as a boundary element separating large chromatin domains or affect the expression of nearby genes along Xi^∆*Xist*^.

Although Pol III is known as the RNA polymerase in charge of the transcription of tRNA and 5 S rRNA, Pol III transcription along tRNA genes is also well known to function as the boundary element separating heterochromatin and euchromatin in yeast^19^,^20^. In mammalian cells, accumulating evidence are revealing a versatile role of Pol III^8^. The roles of Pol III transcription in regulating chromatin configuration in mammalian cells are also emerging^19^,^21^. In the mouse, Pol III mediated barrier activity has been observed on Alu^22^ and B2^23^. Genome-wide Hi-C analysis suggests SINE elements are enriched at the boundaries of chromatin topological domains^24^. The *Xist* RNA coating is more concentrated along the SINE-rich DNA regions^14^,^15^. In yeast, certain genomic loci are bound by TFIIIC in the absence of Pol III^25^. They are named as “extra TFIIIC loci (ETC) and are involved in higher-order chromatin organization^26^. Thousands of ETC sites are found in the human genome^27^. TFIIIC and ETCs are interesting subjects for future research. Generating a stable knockdown cell line of Pol III will cause strong side effects, since Pol III is in charge of the transcription of many house-keeping genes, such as tRNAs. To manipulate Pol III transcription, most of the experiments in this study rely on the Pol III inhibitor. Focusing on TFIIIC or a well-characterized binding site of TFIIIC in the genome may reveal more insights. Taken together, these evidences suggest Pol III transcription is involved in regulating chromatin configuration in mammals.

SINE is the major difference of Pol III genes between yeast genome and mammalian genomes. As Pol III genes, SINEs may be involved in regulating chromatin configuration of mammalian genomes. ~1 million copies of Alu occupy ~10% of the human genome^6^. As a retrotransposon, the majority of SINEs are transcriptionally silenced^28^. However, SINEs are non-autonomous retrotransposon. The transcription activity of a SINE cannot directly invoke its transposon activity. By analyzing ENCODE transcriptomes of human cell lines, it is estimated that ~1300 Alu elements are transcriptionally active^28^. Besides the expected Pol III targets, such as tRNA genes, ChIP-seq analysis on human cells also reveals many additional Pol III loci near SINEs^27^. Available evidence on XCI also suggests the roles of SINEs in regulating chromatin configuration. During XCI, the X-linked intergenic regions, guided by *Xist* RNA, first form a repressive compartment at the core region of the chromosome territory, while the actively transcribed X-linked genes are located at the periphery of the territory^29^. When a gene is silenced, the gene body is relocated into the silenced core compartment. SINEs, concentrated in the gene-rich regions, may be involved in this process. All these evidences suggest SINE is a special group of Pol III gene involved in regulating chromatin configuration in mammals.

In conclusion, our study suggests that Pol III transcription is involved in XCI and functions as a general mechanism regulating chromatin configuration in mammals.

## Experimental Procedures

### Cell lines and culture

Mouse fibroblast cells were cultured in DMEM medium with 10% FBS at 37 °C in a 5% CO_2_ incubator. The 2loxT cell line is a female mouse fibroblast cell line containing X chromosomes from two different mouse strains: the 129 strain and the *Mus musculus castaneus* (CAST/Ei) strain. The X^129^ allele in 2loxT cells was genetically engineered by inserting two loxP sites into the *Xist* gene body^5^. From the 2loxT cells, 6 mutant female fibroblast cell lines, in which the *Xist* gene was conditionally and allele-specifically deleted from the Xi were generated by transient expression of Cre followed by limiting dilution as previously described^3^. Three female mouse ES cell lines, EL16.7^30^, 3F1^17^ and LS2, with the genotype Xa^129^Xa^Cast^ were cultured in 2i medium with Lif 31. LS2 was in-house derived from blastocysts using 2i medium with Lif.

### Padlock probe library design

The mouse chromosome X DNA sequence (mm_ref_MGSCv37_chrX.mfa.gz) was downloaded from NCBI (ftp://ftp.ncbi.nih.gov/genomes/M_musculus/CHR_X/). The mouse genome annotation (knowngene.txt.gz) was obtained from the UCSC genome annotation database (http://hgdownload.cse.ucsc.edu/goldenPath/mm9/database/). The mouse SNP data (20110602-final-snps.vcf.gz) was obtained from the database of Wellcome Trust Sanger Institute (ftp://ftp-mouse.sanger.ac.uk/current_snps/).

Criteria used in SNP selection and padlock probe design were the following. (1) The SNP data file from Wellcome Trust Sanger Institute carried the SNPs from 17 different mouse strains including *Castaneus* and three substrains of “129”. Only the SNPs from the three substrains of 129 and the *Castaneus* strain were selected. (2) Only the SNPs with ATG (above threshold genotype) score 0 or 1 were selected. SNPs with ATG score 1 or 0 are confirmed SNPs with high genotype quality calls^32^,^33^. (3) Only X-linked SNPs located within gene coding regions were selected. (4) In some cases, two SNPs are located closely. One SNP may interfere the binding of the extension arm or the ligation arm of the padlock probe targeting the other SNP (the “shoulder” effect). Such SNPs were excluded. (5) SNPs located too close to the exon boundaries generate inconvenience for designing the padlock probe. Such SNPs were excluded. (6) SNP selection and padlock probe design were further optimized by a computer program called “ppdesigner”^34^, which takes into consideration factors such as the melting temperature (Tm) of the probe and the secondary structure of the DNA template.

To design the padlock library to detect transcription from non-coding regions, only the SNPs from introns and intergenic regions were selected. “ppdesigner” was then used to select the pool of SNPs suitable for padlock capture, from which, one padlock probe targeting a SNP along the sense strand of X chromosome and one padlock probe targeting the antisense strand were randomly selected from every 5 kb region. Two closely located SNPs with the “shoulder” effect were excluded. If a 5 kb non-coding region did not contain SNPs suitable for padlock capture, the region was excluded from the detection of the padlock library.

Detailed information about padlock probe design, padlock library amplification, padlock SNP capture and Illumina sequencing are described in Supplemental Experimental Procedures.

### Data analysis

The Solexa sequencing reads were aligned to the mouse genome (mm9) using bowtie2 (version 2.1.0)^35^. We used the default alignment setting except that gaps were disabled. Reads with single best alignment position along the X chromosome were selected for further data analysis. For each SNP, sequencing reads carrying SNPs different from the known SNPs for the 129 allele and the *Castaneus* allele were considered as sequencing errors. The sequencing error rate was calculated for each SNP, and the sequencing error was deducted from the allelotyping data of the SNP. A SNP was considered “undetected” if the reads count is less than 10. The data for all SNPs belonging to one UCSC Known Gene ID^36^ were combined. Under the annotation system of UCSC Known Gene, one gene name may possess multiple gene IDs referring to different splicing isoforms or alternative transcription start sites of the gene. To identify the reactivated genes along Xi^∆*Xist*^, the data for all gene IDs under one gene name were then combined. Genes carrying no read count for both the 129 allele and the *Castaneus* allele were considered un-allelotyped and were removed from the data set. To prevent dividing by zero, a pseudo-count of 10 was given to each reads count. To identify reactivated genes, we compared each Xi^129-1lox^Xa^Cast^ cell line with the control cell line (Xi^129^Xa^Cast^). A gene’s allelotype was calculated as “Reads^129/^Reads^Cast^”. For a reactivated gene, its allelotype in the mutant sample should be at least 3 folds of its allelotype in the control. Moreover, a reactivated gene should be biallelically expressed in the mutant cells (0.01 < allelotype < 100). After a gene’s allelotype was analyzed, its UCSC Known Gene ID was used in downstream data analysis, for example, analyzing the distribution of SINEs upstream and downstream of the gene’s transcription start site. If a gene name possesses multiple UCSC Known Gene IDs, one gene ID was computationally selected in a random manner to represent the gene. All gene IDs provided in the manuscript are the gene IDs used in the downstream data analysis.

To analyze the noncoding-region SNP capture data, we defined two terms “allelotype” and “expression pattern”. The allelotype of a given SNP refers to its allele-specific expression status in a certain cell type. The allelotype of a SNP could be 129-specific, cast-specific, biallelic, undetected or undetermined. To determine whether the non-coding transcription across a given SNP is silenced by *Xist*, the allelotype of the SNP from three cell types (Xa^129^Xa^Cast^, Xi^129^Xa^Cast^ and Xi^129-1lox^Xa^Cast^) were compared to determine the “expression pattern” of the SNP (Fig. 3D). The expression pattern of a SNP could be “silenced”, “reactivated” or “non-*Xist* regulated”. Four parameters were used in data analysis: detection threshold, pseudo-count, r and R. If a SNP’s reads count from a given allele was less than or equal to 10, the expression of the SNP from the allele was considered “undetected” and the reads count is set to 0 (detection threshold = 10). If a SNP’s expression from both alleles was undetected, the allelotype of the SNP was considered as “undetected” and the SNP was excluded from further data analysis. To determine the allelotype of a SNP, we calculated r (r = Reads^129^/Reads^Cast^, Fig. 3C). To prevent dividing by zero, a pseudo-count of 10 was given to each reads count (pseudo-count = 10). To determine a SNP’s expression pattern, we calculated R = r_e/_r_c_, where r_e_ is the r of Xi^129-1lox^Xa^Cast^ cell type and r_c_ is the r of Xi^129^Xa^Cast^ cell type. The thresholds of r and R used in data analysis are shown (Fig. 3C and D). To avoid the subjectivity in threshold choice, the data was analyzed using a different set of thresholds and the results are shown in Supplementary Figure S4. Venn diagrams were drawn by Venny (http://bioinfogp.cnb.csic.es/tools/venny/).

### Pol III inhibition

RNA polymerase III inhibitor (Merch Millipore, Cat# 557403) was used at the concentration of 30 μM in all experiments. For quantitative RT-PCR and padlock capture, 2loxT.S2 cells were treated with the inhibitor for 4 hrs in cell culture medium containing 2% FBS and 2 mM BrU (Sigma-Aldrich, Cat#850187). Total RNA was isolated by TRIzol (Life technologies). BrU-labeled RNA was isolated as previously described^37^. cDNA was synthesized using SuperScript II Reverse Transcriptase kit (Invitrogen, Cat#18064-014). The real-time PCR was carried out on the CFX Connect real-time PCR system (Bio-Rad) using the SsoAdvanced Universal STBY Green Supermix (Bio-Rad). The following PCR primers were used: SNP35888319P1 (5′-TAACATAAATAAGTATCTCTACATCACTTACAATACCTGAGA-3′); SNP35888319P2 (5′-AATATTGCTGCCTCAGCTGGTAGCT-3′); SNP35888319P3 (5′-ACATGTCCCTACAGACAGTGAATGCAT-3′); SNP35888319P4 (5′-CCCATGGGCTTGCTCTTCCCC-3′); SNP35888319P5 (5′-TCTCAAACATGCCCATGGGCTT-3′); tRNALeuF (5′-CGCCAGACTCAAGCTATGGC-3′); tRNALeuR (5′-TGTCAGAAGTGGGATTCGAACC-3′); tRNATyrF (5′-CCTTCGATAGCTCAGTTGGTAGAG-3′); tRNATyrR (5′-GGATTCGAACCAGCGACCTAAGGATATC-3′); SNP147087190F (5′-AGCAGCAGGCAGAAGCCAGGACT-3′); SNP147087190R (5′-AGTCAGCGGTGGAAACACAGTT-3′); SNP8441359F (5′-CTATGGGACCCACAGCAGGC-3′); SNP8441359R (5′-ACAGCCCTGTTGAGAGGCCCTCTCC-3′); SNP12807658F (5′-AAACCCTCCCCCTTCAAGCCC-3′); SNP12807658R (5′-AATGGATATTGTCACTTTGCGTGAATTGTGC-3′); SNP98673711F (5′-ATACTGTGTGCAGAAAAGCCTGCTGA-3′); SNP98673711R (5′-AGGGGAGCAGCCTTCAGGCCATACA-3′); SNP12344545F: 5′-GTAGCTCAGGCGAGAAGCACCTGC-3′; SNP12344545R: 5′-GGATGGCACACACAGCCTTTCAGC-3′; GSPNC545_sqz:5′-CTACTTTGTGATCCGTTGGAAGGCAGGAT-3′. tRNALeuR, tRNATyrR, SNP147087190F, SNP35888319P4, SNP8441359R, SNP12807658R, SNP98673711F, GSPNC545_sqz were used as gene specific primers in reverse transcription.

### Chromosome conformation capture carbon copy (5C)

The 5C experiments were carried out following the protocol described in the previous publication^38^. The 5 C primer library was designed using a series of in-house written perl scripts. 190 primers were designed to target an ~1 mb region (mm9 ChrX: 70,831,000-71,821,000). The following primers were used to amplify 5 C ligation products: 5C-F (5′-AATGATACGGCGACCACCGAGATCTACACGCTACACGCCATTAACCCTCACTAAAGGGA-3′); 5C-R-index1 (5′-CAAGCAGAAGACGGCATACGAGATGATCTGCGGTC TGCCATCCGCCCTATAGTGAGTCGTATTA-3′); 5C-R-index2 (5′-CAAGCAGAAGACGGCATACGAGATTGGTCACGGTCTGCCATCCGCCCTATAGTGAGTCGTATTA-3′); 5C-R-index3 (5′-CAAGCAGAAGACGGCATACGAGATCACTGTCGGTCTGCCATCCGCCCTATAGTGAGTCGTATTA-3′); 5C-R-index4 (5′-CAAGCAGAAGACGGCATACGAGATATTGGCCGGTCTGCCATCCGCCCTATAGTGAGTCGTATTA-3′). For data analysis, the data from each sample was normalized according to its sequencing depth. Heat maps were generated by Java Treeview^39^. The heatmaps were binned 4 × 4 by taking the median. To generate the subtractive heatmap, the interaction count of the Pol III inhibition sample was subtracted by the interaction count of the control sample for each interaction. The resulting data matrix was binned 4 × 4 by taking the median. The data matrix was then converted to the logarithm of the subtractive interaction. To avoid taking the logarithm of zero, a pseudocount of 1 was given to a subtractive interaction with a value of zero. For a negative subtractive interaction, the logarithm was taken on its absolute value and the resulting value was assigned as negative.

### shRNA knockdown of TFIIIC

An shRNA system (OligoEngine, pSUPER RNAi System) was used to knockdown mouse general transcription factor IIIC, polypeptide 4 (Gtf3c4, gene ID: 269252). DNA oligos containing the desired shRNA sequence: 84573 F (5′-GATCCCCGCCCAGCTCTTTAATATGTAATTCAAGAGATTACATATTAAAGAGCTGGGCTTTTTA-3′); 84573 R (5′-AGCTTAAAAAGCCCAGCTCTTTAATATGTAATCTCTTGAATTACATATTAAAGAGCTGGGCGGG-3′).

### Allele-specific RT-PCR

Allele-specific RT-PCR was carried out following the protocol described in the previous publication^40^. The restriction enzyme used is StuI (New England Biolabs, R0187S). Primers used to amplify Med14: Sqz893StuIF (5′-AGAATGGCCTTCAGTTCCTGAGGC-3′); Sqz893StuIR: 5′-GAAGATTGACCACTTATCAATAGA-3′); Southern blotting oligo probe: Sqz893-sprobe (5′-CTGGTGGGCTCTTGCGTGTACACTG-3′).

### Data Availability

The sequencing data of this study is available in sequence read archive (SRA, accession number SRP075994).

## Additional Information

**How to cite this article:** Hong, R. *et al*. High-resolution RNA allelotyping along the inactive X chromosome: evidence of RNA polymerase III in regulating chromatin configuration. *Sci. Rep.* **7**, 45460; doi: 10.1038/srep45460 (2017).

**Publisher's note:** Springer Nature remains neutral with regard to jurisdictional claims in published maps and institutional affiliations.

## Supplementary Material

Supplementary Materials
