# Supplementary material for: High-resolution RNA allelotyping along the inactive X chromosome: evidence of RNA polymerase III in regulating chromatin configuration
Source: Sci Rep. 2017 Apr 3;7:45460. doi: 10.1038/srep45460 (PMC5377358; doi:10.1038/srep45460)
Supplement: Supplementary Materials [file srep45460-s1.doc]

# High-resolution RNA allelotyping along the inactive X chromosome: evidence of RNA polymerase III in regulating chromatin configuration

Ru Hong^1^, Bingqing Lin^1,5^, Xinyi Lu^3^, Lan-Tian Lai^1^, Xin Chen^2^, Amartya Sanyal^1^, Huck-Hui Ng^3^, Kun Zhang^4^ and Li-Feng Zhang^1,*^

^1^School of Biological Sciences, Nanyang Technological University, 60 Nanyang Drive, Singapore 637551.

^2^Division of Mathematical Sciences, School of Physical and Mathematical Sciences, Nanyang Technological University, 21 Nanyang Link, Singapore 637371.

^3^Genome Institute of Singapore, Singapore 138672.

^4^Department of Bioengineering, University of California at San Diego, La Jolla, CA 92093, USA.

^5^Current address: Institute of Statistical Science, Shenzhen University, Shenzhen 518060, China.

*Correspondence: zhanglf@ntu.edu.sg

**Supplemental Figures**

**
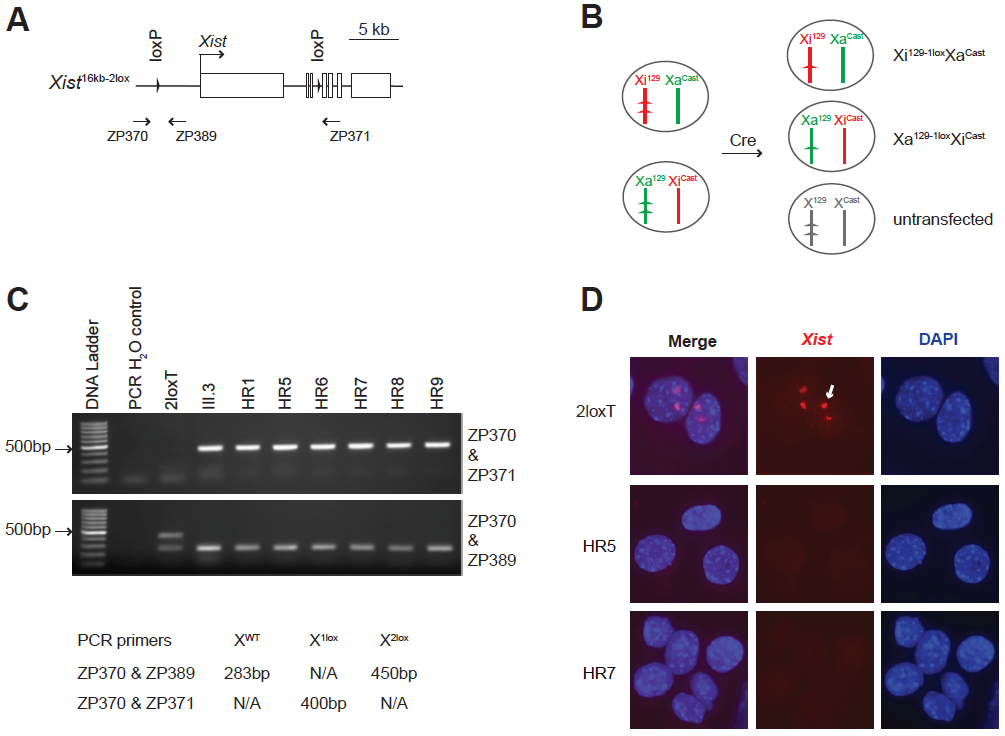
**

**Figure S1.** **Generation of the mutant fibroblast cell lines, in which the *Xist* gene was allele-specifically deleted from the Xi.** (A) The structure of a mutant *Xist* allele (the 2lox allele), in which two loxP sites were inserted into an X chromosome of the 129 genetic background to flank a 16kb region of the *Xist* gene body^1^. (B) Genotypes of X^129-2lox^X*^Castaneus^* fibroblast cells (2loxT cells) before and after transient expression of Cre. (C) PCR genotyping results on six X^129-1lox^X^Cast^ cell lines (HR1, 5-9) established. III.3, an Xi^129-1lox^Xa^Cast^ cell line established in a previous study ^2^, was used as a control cell line for PCR genotyping. (D) *Xist* RNA FISH on established cell lines to confirm that the *Xist* gene deletion occurred on Xi. The results from two representative cell lines (HR5 & HR7) are shown. The white arrow is pointing to an *Xist* RNA cloud signal. Note: the 2loxT cell line is a transformed cell line, in which many cells are tetraploid. Therefore, one or more *Xist* clouds can be seen in each nucleus.


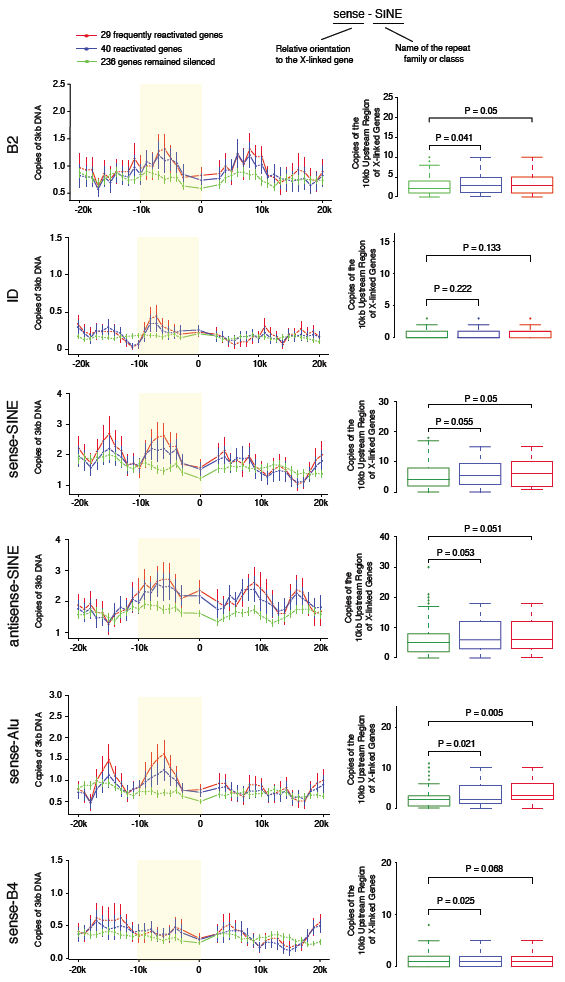


**Figure S2. Distribution of SINEs along the X-linked genes.** The 20 kb upstream and the 20 kb downstream regions of each X-linked gene were scanned by a sliding window of 3 kb with a step size of 1 kb. The average element copy number of a 3 kb intronic region of a gene is used to represent the density of the element within the gene body. Data are shown as mean ± S.E.M. Box plots are element copy numbers of the 10 kb DNA region located 10 kb upstream of X-linked genes.

**
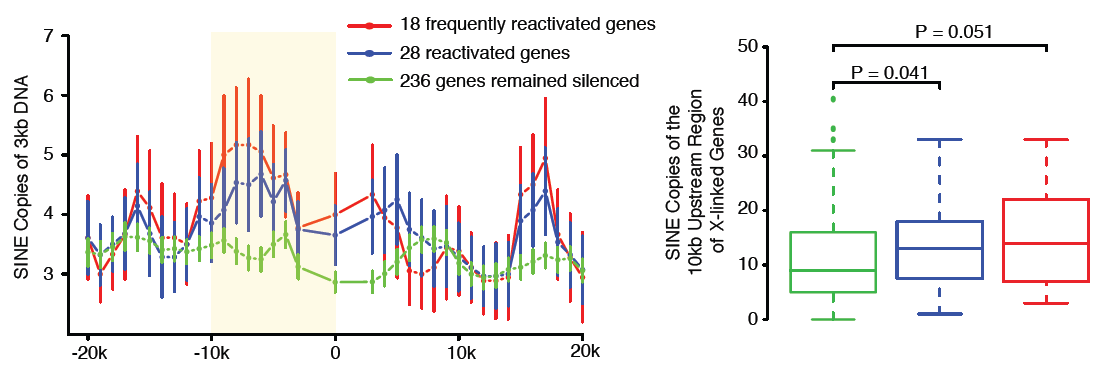
**

**Figure S3. Distribution of SINEs along the X-linked genes.** Constitutive escapees of XCI and genes biallelically expressed from the control cell line (Xi^129^Xa^Cast^) were removed from the list of reactivated genes. The 20 kb upstream and the 20 kb downstream regions of each X-linked gene were scanned by a sliding window of 3 kb with a step size of 1 kb. The average copy number of a repetitive element within a 3 kb intronic region of a gene is used to represent the density of the repetitive element within the gene body. Data are shown as mean ± S.E.M. Box plots show the of copy number of a repetitive element within the 10 kb DNA region upstream of X-linked genes.


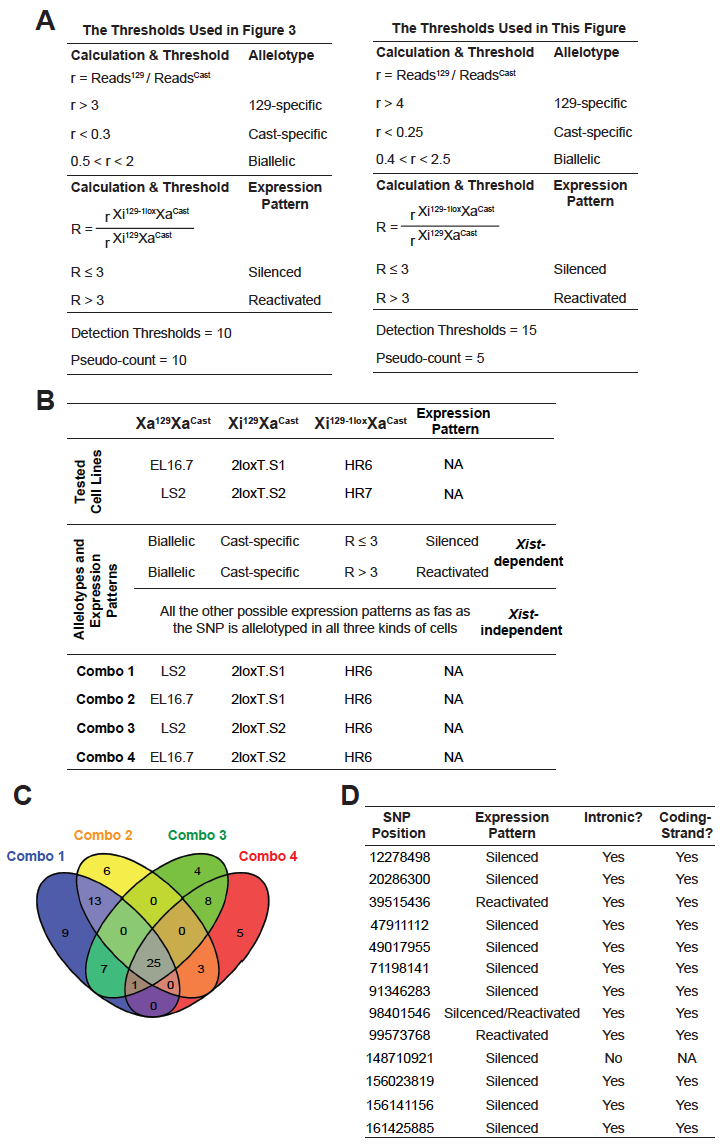


**Figure S4. Transcription from non-coding regions is largely independent from *Xist*-mediated transcriptional silencing (data analysis using different thresholds).** (A) The thresholds used in generating the data shown in Figure 3 and the new thresholds used in this Figure. (B) Definitions of “allelotype” and “expression pattern” used to describe the allele-specific expression profile of an X-linked non-coding SNP. Cell lines of three different genotypes were used in the study. Two cell lines were selected for each genotype. The data from different cell lines were formed in different combinations to test the consistency of the experiments. (C) A Venn diagram showing the non-coding SNPs with *Xist*-independent expression patterns from the data analysis of four different combinations of data sets. (D) Non-coding SNPs with *Xist*-dependent expression pattern from the data analysis of four different combinations of data sets.

**
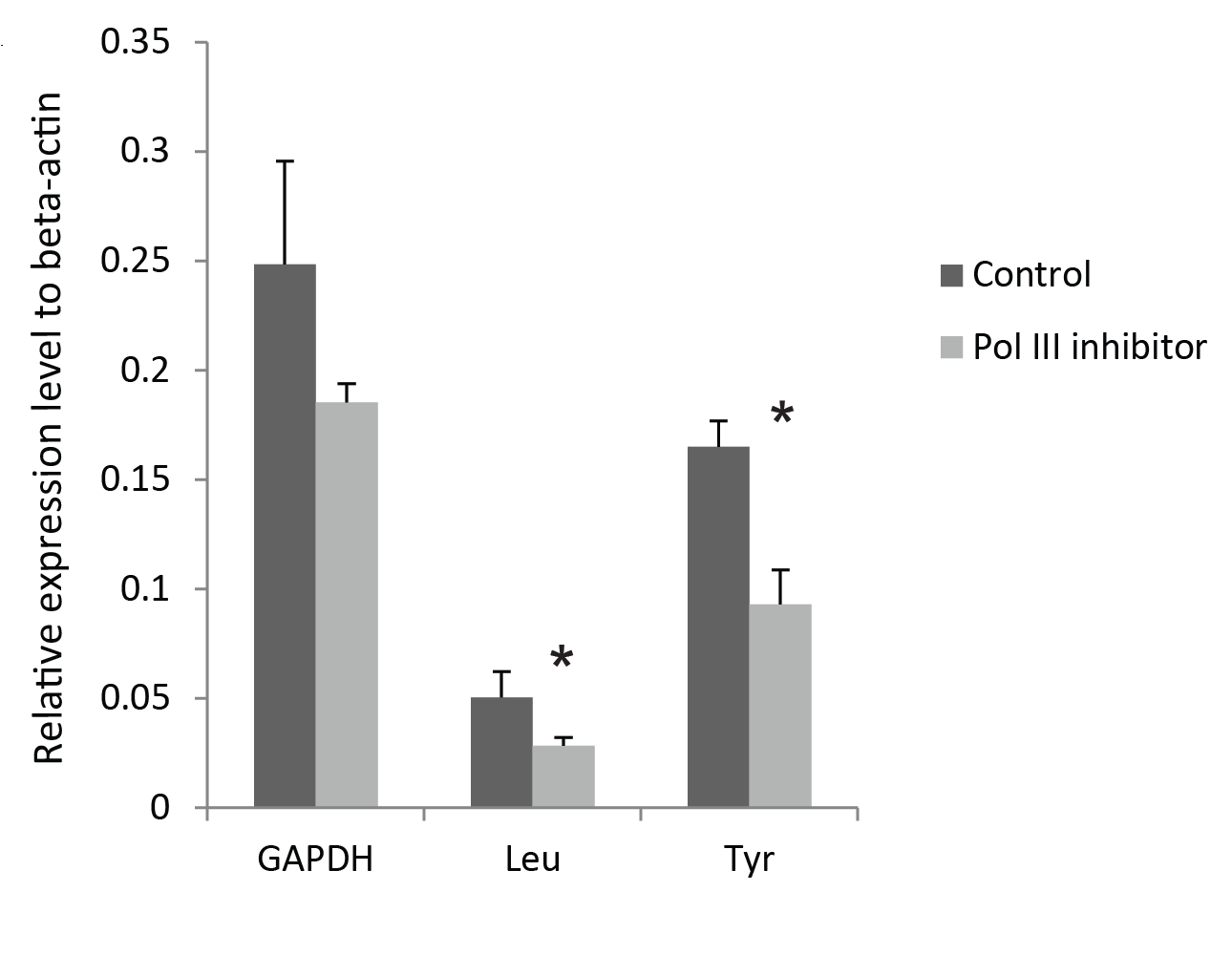
**

**Figure S5. The expression levels of *Gapdh* and tRNA precursor upon Pol III inhibition.** 2loxT.S2 cells were treated with a Pol III inhibitor. Quantitative RT-PCR was carried out to check the expression level of *Gapdh* and two tRNA precursors (Leu and Tyr). The expression levels were normalized with *beta-actin*. Data are shown as mean ± S.E.M of biological triplicate. The statistical analysis used is the Student’s *t*-test. One asterisk indicates *P*-values smaller than 0.05.

**
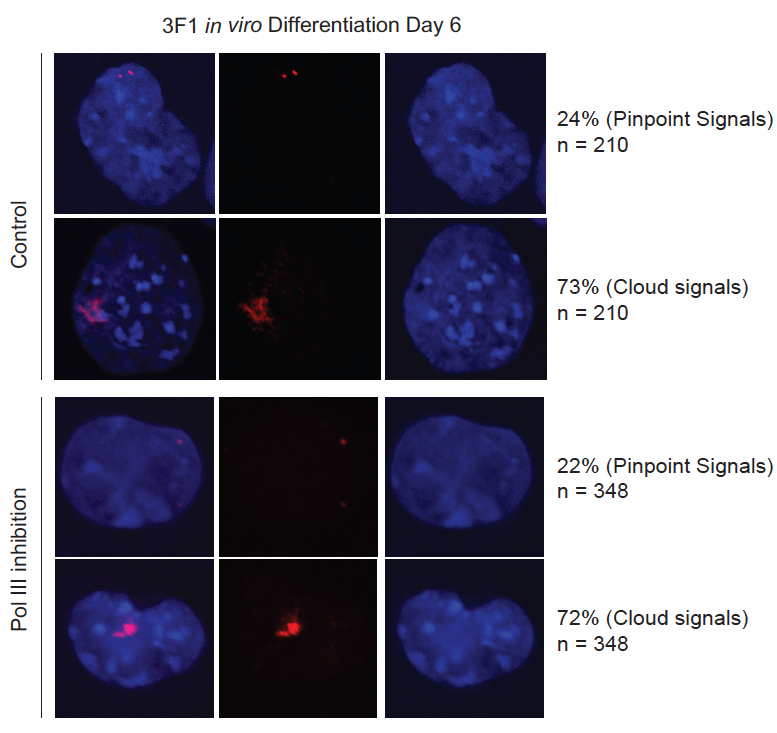
**

**Figure S6. Xist RNA FISH.** 3F1 cells were differentiated *in vitro* for 6 days. Pol III inhibition was carried out during the first two days of *in vitro* differentiation. *Xist* RNA was detected by a Cy3-labeled nucleotide probe (red). DNA was stained by DAPI (blue).

**Supplemental Tables**

**Table S1. Genes reactivated or derepressed along Xi^∆^*^Xist^*.**

| No. | **Gene Name** | **UCSC Known Gene** | **Allelotype (Control)** | **Average Allelotype (Mutants)** | **Fold Increase of Allelotype in Mutants** | No. | **Gene Name** | **UCSC Known Gene** | **Allelotype (Control)** | **Average Allelotype (Mutants)** | **Fold Increase of Allelotype in Mutants** |
| --- | --- | --- | --- | --- | --- | --- | --- | --- | --- | --- | --- |
| 7 | Pls3 | uc009tqk.2 | 0.00 | 0.49 | 1178.5 | 3 | Kdm6a | uc009sso.1 | 0.01 | 0.81 | 129.0 |
| 6 | Eif2s3x | uc009ttd.1 | 0.06 | 2.45 | 41.6 | 2 | Snx12 | uc009tww.2 | 0.16 | 0.71 | 4.4 |
| 5 | Fmr1 | uc009tiy.1 | 0.14 | 5.88 | 43.4 | 2 | Gpkow | uc009sma.1 | 0.00 | 0.23 | 84.0 |
| 5 | Med14 | uc009srd.1 | 0.00 | 1.06 | 1286.9 | 2 | Vbp1 | uc009tqd.1 | 0.00 | 0.18 | 2510.7 |
| 5 | Ddx3x | uc009srl.2 | 0.12 | 10.48 | 89.0 | 2 | Zdhhc9 | uc009tbz.1 | 0.00 | 0.10 | 282.2 |
| 5 | Utp14a | uc009tcb.1 | 0.00 | 1.78 | 1029.3 | 2 | Kdm5c | uc009uqd.2 | 0.43 | 1.56 | 3.6 |
| 5 | 2610029G23Rik | uc009uat.1 | 0.00 | 6.08 | 2061.8 | 2 | Timp1 | uc009sty.1 | 0.01 | 0.37 | 51.4 |
| 5 | Sh3kbp1 | uc009uta.2 | 0.00 | 0.40 | 124.0 | 2 | Bcorl1 | uc009tcc.1 | 0.02 | 0.09 | 6.0 |
| 4 | B230206F22Rik | uc009tzv.1 | 0.19 | 0.77 | 4.0 | 2 | Uba1 | uc009stm.2 | 0.00 | 0.50 | 3459.0 |
| 4 | Magee1 | uc009uav.1 | 0.00 | 0.08 | 78.4 | 1 | Usp11 | uc009stp.2 | 0.00 | 0.20 | 194.8 |
| 4 | Zfx | uc009ttb.1 | 0.00 | 0.19 | 109.6 | 1 | Sh3bgrl | uc009uct.1 | 0.00 | 0.06 | 141.7 |
| 4 | Msn | uc009tue.1 | 0.00 | 0.02 | 58.4 | 1 | Plxna3 | uc009too.1 | 0.00 | 0.01 | 15.0 |
| 4 | Ikbkg | uc009toz.2 | 0.15 | 2.38 | 15.8 | 1 | Tmsb15b2 | uc009ujg.1 | 0.00 | 0.03 | 13.0 |
| 4 | Bgn | uc009tlw.2 | 0.00 | 0.12 | 599.3 | 1 | Pcyt1b | uc009tsv.1 | 0.00 | 0.01 | 64.5 |
| 4 | 1810030O07Rik | uc009sqy.1 | 0.19 | 1.81 | 9.4 | 1 | Trmt2b | uc009ufs.2 | 0.01 | 0.12 | 20.6 |
| 3 | Nono | uc009txs.2 | 0.00 | 1.34 | 472.7 | 1 | 6720401G13Rik | uc012hgw.1 | 0.16 | 0.75 | 4.7 |
| 3 | AK170409 | uc009ssq.1 | 0.01 | 1.67 | 171.0 | 1 | Map3k15 | uc009utb.2 | 0.01 | 0.17 | 26.8 |
| 3 | Mid1 | uc009uyf.1 | 0.00 | 0.51 | 246.7 | 1 | Bmi1 | uc009uoe.1 | 0.00 | 0.11 | 96.4 |
| 3 | Figf | uc009uvm.1 | 0.00 | 0.15 | 283.9 | 1 | Iqsec2 | uc009uqb.1 | 0.00 | 0.02 | 110.5 |
| 3 | Fundc1 | uc009ssf.1 | 0.00 | 2.20 | 2077.2 | 1 | Acot9 | uc009urt.1 | 0.00 | 0.02 | 6.6 |

Note: The frequently reactivated genes, which were reactivated in two or more mutant samples, are highlighted in green. The gene names of escapees of XCI are highlighted in red for constitutive escapees^3^ and blue for facultative escapees^4^. “No.” indicates how many times the gene was identified as a reactivated gene among the 8 mutant samples.

**Table S2. Correlation coefficient between the gene reactivation pattern and genomic features including different families of repetitive DNA elements and the *Xist* RNA coating.**

| No. | **Genomic Features** | **Spearman coefficient** | No. | **Genomic Features** | **Spearman coefficient** |
| --- | --- | --- | --- | --- | --- |
| 1 | B2 | 0.65 | 22 | CR1 | 0.28 |
| 2 | Alu | 0.62 | 23 | Y-chromosome | 0.23 |
| 3 | ID | 0.62 | 24 | Genie | 0.23 |
| 4 | B4 | 0.61 | 25 | MER2_type | 0.21 |
| 5 | L2 | 0.57 | 26 | TcMar | 0.21 |
| 6 | MaLR | 0.54 | 27 | Gypsy | 0.19 |
| 7 | *Xist* (RAP) | 0.54 | 28 | LTR | 0.17 |
| 8 | Tc2 | 0.53 | 29 | ERV1? | 0.15 |
| 9 | snRNA | 0.52 | 30 | MIR | 0.14 |
| 10 | tRNA | 0.47 | 31 | Tip100 | 0.09 |
| 11 | MuDR | 0.44 | 32 | srpRNA | 0.08 |
| 12 | ERVL | 0.43 | 33 | Tigger | 0.07 |
| 13 | RTE | 0.43 | 34 | AcHobo | 0.03 |
| 14 | *Xist* (CHART-seq) | 0.41 | 35 | TcMar? | 0.02 |
| 15 | Mariner | 0.38 | 36 | Satellite | 0.01 |
| 16 | MER1_type | 0.38 | 37 | Charlie | -0.06 |
| 17 | hAT | 0.34 | 38 | L1 | -0.14 |
| 18 | rRNA | 0.32 | 39 | ERV1 | -0.27 |
| 19 | scRNA | 0.29 | 40 | Helitron? | -0.31 |
| 20 | Simple_repeat | 0.29 | 41 | ERVK | -0.33 |
| 21 | ERV | 0.29 | 42 | Low_complexity | -0.33 |

**Table S3. The non-coding region SNPs with *“non-Xist regulated”* expression pattern.**

| No. | SNP Position | Strand | No. | SNP Position | Strand | No. | SNP Position | Strand |
| --- | --- | --- | --- | --- | --- | --- | --- | --- |
| 1 | 5,277,117 | + | 30 | 74,771,919 | - | 58 | 130,809,258 | + |
| 2 | 7,227,208 | - | 31 | 75,813,496 | + | 59 | 131,227,063 | - |
| 3 | 7,768,502 | + | 32 | 75,870,843 | - | 60 | 131,304,049 | - |
| 4 | 8,239,774 | + | 33 | 77,780,358 | + | 61 | 133,464,368 | + |
| 5 | 8,845,449 | - | 34 | 85,650,688 | - | 62 | 133,539,347 | + |
| 6 | 12,344,545 | - | 35 | 90,818,736 | + | 63 | 137,356,878 | + |
| 7 | 12,807,658 | + | 36 | 91,423,174 | + | 64 | 146,819,569 | + |
| 8 | 12,879,544 | - | 37 | 92,018,272 | + | 65 | 146,877,146 | + |
| 9 | 13,373,553 | + | 38 | 93,117,207 | + | 66 | 146,974,711 | + |
| 10 | 17,833,105 | - | 39 | 97,175,901 | - | 67 | 147,017,388 | + |
| 11 | 18,519,844 | - | 40 | 98,292,961 | + | 68 | 147,029,398 | - |
| 12 | 19,816,795 | - | 41 | 98,395,512 | + | 69 | 147,087,190 | - |
| 13 | 20,235,745 | - | 42 | 98,673,711 | - | 70 | 147,809,926 | - |
| 14 | 20,435,354 | - | 43 | 99,496,460 | + | 71 | 148,004,349 | - |
| 15 | 20,634,135 | + | 44 | 99,557,757 | + | 72 | 148,468,717 | - |
| 16 | 34,158,892 | + | 45 | 99,595,155 | + | 73 | 148,545,040 | + |
| 17 | 34,169,014 | + | 46 | 100,496,027 | + | 74 | 148,756,047 | + |
| 18 | 35,535,031 | - | 47 | 100,502,644 | + | 75 | 149,833,356 | - |
| 19 | 35,888,319 | + | 48 | 100,580,718 | - | 76 | 151,938,596 | + |
| 20 | 39,428,600 | - | 49 | 101,701,054 | - | 77 | 156,023,004 | + |
| 21 | 39,505,390 | - | 50 | 101,817,822 | + | 78 | 157,237,893 | + |
| 22 | 47,911,112 | + | 51 | 101,857,200 | + | 79 | 157,309,699 | + |
| 23 | 49,502,374 | + | 52 | 102,677,289 | - | 80 | 159,220,820 | + |
| 24 | 51,187,025 | + | 53 | 118,449,334 | + | 81 | 160,380,742 | + |
| 25 | 64,979,833 | - | 54 | 126,884,955 | - | 82 | 165,517,285 | - |
| 26 | 70,691,121 | + | 55 | 126,903,279 | - | 83 | 165,806,562 | - |
| 27 | 71,552,769 | - | 56 | 130,179,355 | + | 84 | 166,173,763 | - |
| 28 | 72,632,962 | - | 57 | 130,452,728 | - | 85 | 166,446,176 | + |
| 29 | 72,966,295 | + |  |  |  |  |  |  |

Note: The mouse chromosome X DNA sequence (mm_ref_MGSCv37_chrX.mfa.gz) was used as the reference genome. The “Strand” column indicates the orientation of the detected transcription. A “+” sign indicates that the detected RNA transcript carries the same sequence as the reference genome. A “-“ sign indicates that the detected RNA transcript carries the complementary sequence of the reference genome.

**Table S4.** X-linked genes affected by Pol III inhibition during XCI in vitro.

| **UCSC Known Gene** | **Gene Name** | **UCSC Known Gene** | **Gene Name** | **Gene ID (UCSC Known Gene)** | **Gene Name** |
| --- | --- | --- | --- | --- | --- |
| uc009utb.2 | Map3k15 | uc009tmd.2 | Pnck | uc009smk.2 | Tcfe3 |
| uc009tua.1 | Zc3h12b | uc009tfj.2 | 1700013H16Rik | uc009txf.1 | Med12 |
| uc009uqe.2 | Kdm5c | uc009tsw.1 | Pcyt1b | uc009smz.1 | Pim2 |
| uc009uqv.1 | Klf8 | uc009srr.1 | Cask | uc009toa.1 | Emd |
| uc012hom.1 | Armcx6 | uc009suj.2 | Zfp182 | uc009tww.2 | Snx12 |
| uc009tnt.1 | Mecp2 | uc009thg.2 | Rbmx | uc009ugm.2 | Armcx1 |
| uc009snm.1 | Gata1 | uc009tnl.1 | Hcfc1 | uc009tnz.1 | Flna |
| uc009uij.1 | Wbp5 | uc009tjy.1 | Mtmr1 | uc009sqq.2 | Bcor |
| uc009stx.2 | Syn1 | uc009snb.1 | Slc35a2 | uc009ter.1 | Hprt |
| uc009uwa.2 | Fancb | uc009sug.2 | A230072C01Rik | uc012hkf.1 | Bgn |
| uc009ulz.2 | Tmem164 | uc009stb.1 | Phf16 | uc009tyt.1 | Phka1 |
| uc009sla.2 | Dgkk | uc012hnd.1 | Tsix | uc009smx.1 | Otud5 |
| uc009upr.1 | Huwe1 | uc012hjj.1 | Fmr1nb | uc009toy.1 | G6pdx |
| uc009tbt.1 | Ocrl | uc009tem.1 | Ccdc160 | uc009tqj.2 | Pls3 |
| uc009txd.1 | Il2rg | uc009tfd.1 | Cxx1c | uc009tkv.1 | Nsdhl |
| uc009tlq.2 | Zfp275 | uc009tkw.2 | Zfp185 | uc009sub.1 | Cfp |
| uc009teq.1 | Phf6 | uc009tex.2 | Fam122b | uc009tgn.1 | Fhl1 |
| uc009stq.3 | Usp11 | uc009uav.1 | Magee1 | uc009smr.1 | Gripap1 |
| uc009sne.1 | Timm17b | uc009tvk.1 | Pja1 | uc009suu.1 | Klhl13 |
| uc009tbu.1 | Apln | uc009spi.2 | B630019K06Rik | uc009tvf.1 | Yipf6 |
| uc009usz.2 | Sh3kbp1 | uc009twp.2 | Dlg3 | uc012hkh.1 | Slc6a8 |
| uc009txt.2 | Nono | uc009sme.1 | Praf2 | uc012hmu.1 | Kif4 |
| uc012hfg.1 | Elk1 | uc009sni.2 | Hdac6 | uc009tmb.1 | Dusp9 |
| uc009ste.2 | Ndufb11 | uc009spy.1 | Dynlt3 | uc009uun.2 | Syap1 |
| uc012hgw.1 | 6720401G13Rik | uc009tzz.1 | Rlim | uc009tre.2 | Tmem47 |
| uc009snf.2 | Pcsk1n | uc009tue.1 | Msn | uc009teu.1 | Plac1 |
| uc009tyf.1 | AK033245 | uc009soo.1 | Ftsj1 | uc009sxp.1 | Akap17b |
| uc009sso.1 | Kdm6a | uc009tyk.2 | Ercc6l | uc009uow.1 | Fgd1 |
| uc009tcc.1 | Bcorl1 | uc009sle.2 | Clcn5 | uc009tnp.2 | Irak1 |
| uc009sss.1 | Chst7 | uc009soi.1 | Ebp |  |  |
| uc009skz.1 | Shroom4 | uc009sng.1 | Eras |  |  |
| uc009tgh.2 | Ddx26b | uc012hjl.1 | Ids |  |  |

Note: The gene names of escapees of XCI are highlighted in red for constitutive escapees^3^ and blue for facultative escapees^4^.

**SUPPLEMENTAL EXPERIMENTAL PROCEDURES**

**Padlock probe design**

The extension arm and the ligation arm of each padlock were 18-20nt in length. The DNA region captured by each padlock probe was 24-26nt. The structure of each synthetic padlock probe was: 5’-aggaccggatcaact-(ligation arm)-cttcagcttcccgatatccgacggtagtgt-(extension arm)-cattgcgtgaaccga-3’. The padlock probe library was synthesized by LC Sciences (USA).

**Padlock library amplification**

The padlock probe library was amplified as previously described^5,6^. In brief, the two primers used in library amplification were eMIP_CA_F (5’-TGCCTAGGACCGGATCAACT-3’) and eMIP_CA_R (5’-GAGCTTCGGTTCACGCAATG-3’). The two nicking endonucleases used were Nt.AlwI R0627S, NEB) and Nb.BsrDI (R0648S, NEB). The PCR amplification was carried out in a real time PCR system (CFX Connect, Bio-Rad) using KAPA SYBR Fast Master Mix (KK4601, KAPA Biosystems).

**Padlock SNP capture**

Padlock SNP capture was carried out as previously described^5,6^. In brief, each reaction was carried out in 20µl volume containing 1 unit Ampligase (A3210K, Epicentre), 1 unit Phusion High-Fidelity DNA Polymerase (M0530, New England BioLabs), 1 x Phusion High-Fidelity DNA Polymerase buffer, 10nM dNTP. For coding-region SNP capture, cDNA was generated using SuperScript III first-strand synthesis system (18080-051, Life Technologies). 200ng single stranded cDNA and 2pmol padlock probe were used in each reaction. For noncoding-region SNP capture, cDNA was synthesized using iScript reverse transcription kit (170-8840, Bio-Rad). 1.2μg single stranded cDNA and 4pmol padlock probe were used in each reaction. Nicotinamide adenine dinucleotide (NAD^+^) was provided in each reaction at a final concentration of 0.5mM.

**Illumina Sequencing**

The multiplexed sequencing libraries were PCR amplified in a real time PCR system (CFX Connect, Bio-Rad) using the following primers: CA-2-RA.Miseq (5’-AATGATACGGCGACCACCGAGATCTATCGGCTACACGCCTATCGGGAAGCTGAAG-3’); CA-2-FA.Indx7Sol (5’-CAAGCAGAAGACGGCATACGAGATGATCTGCGGTCTGCCATCCGACGGTAGTGT-3’); CA-2-FA.Indx45Sol (5’-CAAGCAGAAGACGGCATACGAGATCGTAGTCGGTCTGCCATCCGACGGTAGTGT-3’); CA-2-FA.Indx76Sol (5’-CAAGCAGAAGACGGCATACGAGATAATAGGCGGTCTGCCATCCGACGGTAGTGT-3’); CA-2-FA.Indx91Sol (5’-CAAGCAGAAGACGGCATACGAGATACATCGCGGTCTGCCATCCGACGGTAGTGT-3’); CA-2-FA.Indx92Sol (5’-CAAGCAGAAGACGGCATACGAGATTCAAGTCGGTCTGCCATCCGACGGTAGTGT-3’); CA-2-FA.Indx93Sol (5’-CAAGCAGAAGACGGCATACGAGATATTGGCCGGTCTGCCATCCGACGGTAGTGT-3’). The following sequencing primers were used: Read1.Miseq (5'-ATCGGCTACACGCCTATCGGGAAGCTGAAG-3'); IndexRead (5’-ACACTACCGTCGGATGGCAGACCG-3’);

**Data Analysis**

**Distribution of the *Xist* RNA and different families of repetitive DNA elements along the mouse X chromosome**

The data of *Xist* RNA enrichment along the Xi in mouse lung fibroblast cells was generated by RNA antisense purification (RAP) ^7^. The data file (GSM1141179_MLFXist_vs_Input.W10000_O7500.bigWig) was downloaded from NCBI website (<http://www.ncbi.nlm.nih.gov/geo/query/acc.cgi?acc=GSM1141179>). The data of *Xist* RNA enrichment along the Xi in mouse embryonic fibroblast cells was generated by CHART-seq (capture hybridization analysis of RNA targets) ^8^. The data file (GSM1182875_MEF.xist.comp.bedGraph.gz) was downloaded from NCBI website (<http://www.ncbi.nlm.nih.gov/geo/query/acc.cgi?acc=GSM1182875>).

For different repetitive DNA elements along the mouse X chromosome, we downloaded the RepeatMasker track (chrX-rmsk.txt) from the UCSC website (<http://hgdownload.soe.ucsc.edu/goldenPath/mm9/database/>). To plot the distribution of each repetitive DNA elements along the mouse X chromosome, we calculated the score of each genomic feature in 200kb bins for every 100kb.

To calculate the Spearman correlation coefficient between different genomic features and the gene reactivation pattern along Xi^129-1lox^, we divided the mouse X chromosome into non-overlapping windows at 8 x10^6^ bp resolution.

**Immuno-RNA FISH**

Immuno-RNA FISH was carried out as previously described ^2^. Immunofluorescence was performed using mouse monoclonal antibodies against RNA polymerase II (Pol II) (Santa Cruz, Cat# sc-47701) and RNA polymerase III (Pol III) (Santa Cruz, Cat# sc-48365) with a secondary antibody conjugated with FITC (green). Immunostaining was followed by RNA FISH. The *Xist* RNA was detected with Sx9 probe, a P1 DNA construct containing a 40 kb genomic fragment covering the *Xist* gene. Nucleotide analogs used in probe labeling were Cy3-dUTP (Amersham, Cat# PA53022). Microscopy work was carried out on an Eclipse Ti microscope (Nikon) with a digital camera (Clara Series model C01, Andor).

**References**

1 Csankovszki, G., Panning, B., Bates, B., Pehrson, J. R. & Jaenisch, R. Conditional deletion of Xist disrupts histone macroH2A localization but not maintenance of X inactivation. *Nat Genet* **22**, 323-324, (1999).

2 Zhang, L. F., Huynh, K. D. & Lee, J. T. Perinucleolar targeting of the inactive X during S phase: evidence for a role in the maintenance of silencing. *Cell* **129**, 693-706, (2007).

3 Yang, F., Babak, T., Shendure, J. & Disteche, C. M. Global survey of escape from X inactivation by RNA-sequencing in mouse. *Genome research* **20**, 614-622, (2010).

4 Peeters, S. B., Cotton, A. M. & Brown, C. J. Variable escape from X-chromosome inactivation: identifying factors that tip the scales towards expression. *Bioessays* **36**, 746-756, (2014).

5 Diep, D. *et al.* Library-free methylation sequencing with bisulfite padlock probes. *Nat Methods* **9**, 270-272, (2012).

6 Zhang, K. *et al.* Digital RNA allelotyping reveals tissue-specific and allele-specific gene expression in human. *Nat Methods* **6**, 613-618, (2009).

7 Engreitz, J. M. *et al.* The Xist lncRNA exploits three-dimensional genome architecture to spread across the X chromosome. *Science* **341**, 1237973, (2013).

8 Simon, M. D. *et al.* High-resolution Xist binding maps reveal two-step spreading during X-chromosome inactivation. *Nature*, (2013).
